# Autophagy in Bone Remodeling: A Regulator of Oxidative Stress

**DOI:** 10.3389/fendo.2022.898634

**Published:** 2022-06-30

**Authors:** Chenyu Zhu, Shiwei Shen, Shihua Zhang, Mei Huang, Lan Zhang, Xi Chen

**Affiliations:** ^1^ School of Kinesiology, Shanghai University of Sport, Shanghai, China; ^2^ School of Sports Science, Wenzhou Medical University, Wenzhou, China; ^3^ The Second School of Medicine, Wenzhou Medical University, Wenzhou, China; ^4^ College of Sports and Health, Shandong Sport University, Jinan, China

**Keywords:** autophagy, oxidative stress, osteoblast, osteoclast, osteoporosis

## Abstract

Bone homeostasis involves bone formation and bone resorption, which are processes that maintain skeletal health. Oxidative stress is an independent risk factor, causing the dysfunction of bone homeostasis including osteoblast-induced osteogenesis and osteoclast-induced osteoclastogenesis, thereby leading to bone-related diseases, especially osteoporosis. Autophagy is the main cellular stress response system for the limination of damaged organelles and proteins, and it plays a critical role in the differentiation, apoptosis, and survival of bone cells, including bone marrow stem cells (BMSCs), osteoblasts, osteoclasts, and osteocytes. High evels of reactive oxygen species (ROS) induced by oxidative stress induce autophagy to protect against cell damage or even apoptosis. Additionally, pathways such as ROS/FOXO3, ROS/AMPK, ROS/Akt/mTOR, and ROS/JNK/c-Jun are involved in the regulation of oxidative stress-induced autophagy in bone cells, including osteoblasts, osteocytes and osteoclasts. This review discusses how autophagy regulates bone formation and bone resorption following oxidative stress and summarizes the potential protective mechanisms exerted by autophagy, thereby providing new insights regarding bone remodeling and potential therapeutic targets for osteoporosis.

## 1 Introduction

Bone is constantly being remodeled to maintain the balance of growth and development of the skeletal system (1). Bone remodeling is essential for the formation and maintenance of bone morphology and the repair of damaged bone (2). Physiological bone remodeling requires a balance between bone formation and bone resorption, while the dynamic balance needs coupling of the activities of different bone cells (e.g., osteoblasts, osteocytes, and osteoclasts) (3). Osteoblasts mainly arise by differentiation of bone marrow mesenchymal stem cells (BMSCs) and play an osteogenic role in the regulation of the synthesis, secretion, and mineralization of the bone matrix (4). At the end stage of bone formation, osteoblasts become encapsulated in the bone matrix and mature into osteocytes, which play a crucial role in bone remodeling (5). Osteoclasts, which are the only bone-resorbing cells in the body, are tissue-specific multinucleated macrophages that arise by the differentiation of monocytes or macrophage precursors on or near the bone surface (6). Bone remodeling consists of four primary stages, including bone resorption, recruitment of osteoblasts and BMSCs, osteoblast differentiation, and completion of bone mineralization (7). Dysfunction of any cell type involved in this process can lead to the failure of bone remodeling followed by the development of bone-related diseases, especially osteoporosis (8).

Oxidative stress plays a pivotal role in the regulation of the balance of bone remodeling processes (9), including effects on bone formation and bone resorption. Reactive oxygen species (ROS) induced by oxidative stress can lead to apoptosis of osteocytes and osteoblasts and inhibit bone mineralization and osteogenesis, which combine with unbalanced osteoclast formation to lead to enhanced bone loss and progression of osteoporosis (10, 11). At physiological levels, ROS can act as signaling molecules involved in cellular processes such as differentiation, proliferation, apoptosis, autophagy, and redox signaling (12). In contrast, excessive ROS levels result in damage to lipids, proteins, and DNA, which can ultimately lead to cell death (13).

Autophagy is an essential metabolic pathway for cell survival in case of nutrient or energy deficiencies, oxidative stress, infections, or hypoxia (14). The cytoplasm or organelles of the cell itself are engulfed into vesicles to form autophagosomes, which are then transported to the lysosome for degradation to remove damaged or aging organelles and to maintain the basal cellular homeostasis (15, 16). In response to oxidative stress, autophagy is regulated by the level of ROS resulting from cellular injury, and it supports cell survival by a cytoprotective mechanism that mitigates the damage resulting from the oxidative stress (17). However, excessive accumulation of ROS can also exacerbate cellular damage by dysregulation of autophagy, leading to mitochondrial dysfunction and increased levels of ROS (18). It appears that the interaction between ROS and autophagy is critical for cellular homeostasis. Therefore, the mode of interaction between autophagy and oxidative stress during bone remodeling warrants further elucidation. Here, we reviewed the mechanism of autophagy in response to oxidative stress during bone remodeling and discussed potential therapeutic targets of the autophagy process for osteoporosis.

## 2 Role of Oxidative Stress in Bone Remodeling

Cellular oxidative stress is caused by an imbalance of intracellular redox homeostasis or a relative overload of ROS (19). Mitochondrial are rod-shaped or elongated under normal conditions, whereas under conditions of oxidative stress, the length and density of mitochondria are significantly reduced as they become fragmented, resulting in impaired cellular metabolic function and increased ROS production, and potentially even cell death (20). Oxidative stress is an independent risk factor for postmenopausal, glucocorticoid, and diabetic osteoporosis (20). By impairing bone remodeling as a result of disruption of the coupling of osteoblasts and osteoclasts, oxidative stress-induced ROS may underlie the main cellular mechanism of osteoporosis (21, 22).

### 2.1 Oxidative Stress in Osteoblasts

At physiological levels, ROS help maintain cellular function, whereas uncontrolled levels of ROS are detrimental (23). As osteoblast differentiation requires energy, BMSCs or preosteoblasts undergo a metabolic transformation whereby mitochondrial respiration and ATP production are increased to ensure an adequate energy supply, which is accompanied by an increase in endogenous ROS (24). Additionally, excessive ROS levels reduce osteogenic differentiation in situations of estrogen deficiency, high glucose, diabetes, inflammation, stress, aging, or other pathophysiological factors, which can decrease metabolic enzyme activity or antioxidant production (25, 26). BMSCs cultured long-term *in vitro* exhibit decreased antioxidant capacities and elevated ROS levels, leading to reduction or loss of osteogenic differentiation potential (27). Likewise, hydrogen peroxide (H_2_O_2_)-induced oxidative stress has been shown to inhibit osteogenic differentiation in rat BMSC as measured by reduction in alkaline phosphatase (ALP) activity and Runx2 and ATF4 expression levels (28) (29). In contrast, reduction in the level of oxidative stress in BMSCs enhanced osteogenic function and restored bone mass and bone microarchitecture in ovariectomized rats (30). In addition, signaling pathways triggered by ROS regulate cell proliferation, growth, differentiation, and even apoptosis, thereby affecting the lifespan of osteoblasts. Mitogen-activated protein kinases (MAPKs) such as c-Jun-N terminal kinase (JNK), extracellular signal-regulated kinase (ERK1/2), and p38 are involved in osteoblasts apoptosis (31–33). High levels of ROS activated the JNK signaling pathway, which increases the transcriptional expression of pro-apoptotic genes such as caspase 3, FASL, and caspase 9 (34). Moreover, ROS induced by H_2_O_2_ continuously stimulated the ERK signaling pathway in osteoblasts, which then enhances the expression of Bax and the hyperpolarization of the mitochondrial membrane potential, thereby resulting in cell apoptosis (35).

### 2.2 Oxidative Stress in Osteoclasts

Oxidative stress and the consequent production of ROS promotes osteoclast differentiation and osteoclastogenesis (36). Receptor activator of nuclear factor-κB ligand (RANKL) stimulation has been shown to increase ROS production in bone marrow mesenchymal stem cells (BMMs) through a tumor necrosis factor receptor-associated factor 6 (TRAF6)/RAC1/nicotinamide adenine dinucleotide phosphate oxidase 1 (Nox1) signaling cascade, resulting in enhanced differentiation of osteoclasts. Conversely, exposure to the antioxidant N-acetylcysteine (NAC) has been shown to inhibit the response of BMMs to RANKL, involving ROS production, activation of the MAPK pathway, and osteoclastogenesis (37). Likewise, in the glucose-induced diabetic osteoporosis model in rats, increased ROS production in osteoclasts and subsequently enhanced expression of proteins related to MAPKs [phosphorylated (p)-ERK, p-JNK, and p-p38], NF-κB (NF-κB, p-IκB, and IKK), and NACHT-LRR-PYD domains-containing protein 3 (NLRP3)-related protein expression, which promotes osteoclast differentiation and bone resorption, were observed (38).

ROS production not only directly enhances osteoclast differentiation but also interacts with osteoblasts to regulate the formation and differentiation of osteoclasts. OPG/RANK/RANKL form a molecular triad that links osteoblasts and osteoclasts and thus plays a significant role in osteoclastogenesis (39, 40). High levels of H_2_O_2_-induced ROS in osteoblasts (including osteoblast-like MG63 cells and primary mice osteoblasts) and BMSCs have been shown to stimulate the expression of RANKL mRNA and protein through ERKs and the PKA-CREB pathway (41). Co-culture of osteoblasts with osteoclast precursor cells has revealed that ethanol (EtOH)-induced RANKL expression depends on intracellular ROS stimulation by NADPH oxidase activity in osteoblast, which promotes osteoclast differentiation (42). These results demonstrate that ROS can promote RANKL secretion by osteoblasts, thereby regulating osteoclast differentiation, thus providing novel insights into the role of ROS production in the regulation of osteoblast-osteoclast communication.

Taken together, these findings suggest that ROS can inhibit osteoblast differentiation and hence also bone formation, in addition to promoting osteoclast differentiation and osteoclastogenesis. The effect of oxidative stress on different cell types and their communication are thought to play an essential role in the development of osteoporosis.

## 3 Role of Autophagy in Bone Remodeling

### 3.1 Autophagy in Osteoblasts

Autophagy plays a significant role in bone formation, including differentiation of BMSCs into osteoblasts to osteocytes, osteogenesis, differentiation, and the formation of bone matrix. BMSC differentiation requires energy, while the products of autophagosomal degradation can be oxidized by mitochondria to provide a suitable energy supply for their differentiation (43). Optimal differentiation of MSCs into osteoblasts involves an early stage of AMP-activated protein kinase (AMPK)/mTOR signaling axis-mediated autophagy as well as a later stage of Akt/mTOR signaling axis activation (44). Conversely, reduction of the level of autophagy directly inhibits the function of endogenous BMSCs and further promotes the development of osteoporosis (45). When MSCs are fully differentiated into osteoblasts, basal autophagy is completely inhibited, but this does not indicate that the differentiated cells are no longer capable of autophagy (46).

Mesenchymal-derived osteoblasts, which are recognized as specialized mineralizing cells in bone formation, are known to play a critical role in the synthesis, secretion, and mineralization of the bone matrix (47, 48). A previous study *in vitro* found that autophagy defects induced by ablation of FIP200 in osteoblasts led to the dysfunction of osteoblasts differentiation (49). Furthermore, downregulation of the expression of autophagy markers, such as LC3-II and ATG7, has been shown to result in the inhibition of osteoblast differentiation (50, 51). The early stage of osteoblast differentiation requires the activation of AMPK, and the terminal stage is dependent on downregulation of AMPK (52, 53), which is mediated by stimulation of AKT and mTOR (54), and then activates cell autophagy.

Additionally, autophagy is also directly involved in the mineralization process of osteoblasts. Conditional knockdown of ATG7 in osteoblasts led to a reduction of mineralization capacity *in vivo* (55), and knockout of the autophagy-related genes Fip200 or Atg5 in Osterix-Cre transgenic mice also resulted in impaired mineralization and reduced bone mass in mice (56). These results indicated that autophagy is required in the mineralization process of osteoblasts, which can be attributed to autophagic vesicles acting as carriers for the secretion of apatite crystals to the extracellular matrix (55).

### 3.2 Autophagy in Osteoclasts

Osteoclasts, which differentiate from hematopoietic mononuclear stem cells in the bone marrow, are critical at the beginning of bone remodeling by bone resorption *via* following differentiation into multinucleated osteoclasts which then migrate to the surface of the bone (57, 58). HIF1-α, which is produced in response to hypoxic stress, has been reported to upregulated BNIP3, which increases the level of Beclin-1 and then activates autophagic flux accompanied by the autophagy-related genes ATGs, thereby leading to increased osteoclastogenesis by upregulation of CTSK, NFATC1, and MMP9 (59). Another study showed that a microgravity environment (rotary cell culture system) increased autophagy in osteoclasts, which then stimulated osteoclast differentiation and osteoclastogenesis (60). Moreover, the level of autophagy initiation protein Beclin-1 has been reported to increase during osteoclast differentiation. Ctsk-cell expression conditional Beclin-1 deficient mice exhibited an increase in the thickness of cortical bone *via* attenuated osteoclast function, while overexpression of Beclin-1 in osteoclast precursors has been reported to enhance autophagy-induced osteoclastogenesis *in vitro* and increase bone resorption (61). Mechanistically, it was concluded that TRAF6-mediated K63-linked ubiquitination at Beclin1-K117 is needed for RANKL-induced osteoclast differentiation (61, 62). These findings support the notion that autophagy in osteoclasts is susceptible to environmental factors such as hypoxic stress and microgravity, which results in further regulation of the differentiation of osteoclasts and osteoclastogenesis.

In addition to its role in osteoclast differentiation, autophagy has also been demonstrated to be essential in osteoclast function. Terminally differentiated osteoclasts are tightly attached to the bone surface by pedicles. F-actin, talin, vinculin, and α-actinin are the key anchor targets for osteoclast attachment, and lysosomes then migrate to the bone surface and resorbing bone (57). The autophagy-related proteins ATG4B, ATG5, ATG7, and LC3 have all been shown to play crucial roles in promoting bone resorption activity. For example, knockdown of ATG5 and ATG7 in osteoclasts has been shown to significantly reduce the depth and volume of bone traps and reduce the ability to deliver lysosomes to the fold membrane boundary, although this does not appear to affect osteoclast formation. The lysosomal secretory function requires ATG5-ATG12 coupling to facilitate LC3 binding to phosphatidylethanolamine. ATG5 deficiency inhibits LC3II production as well as CTSK localization (63, 64).

## 4 Regulation of Autophagy in Oxidative Stress

**Figure 1:**
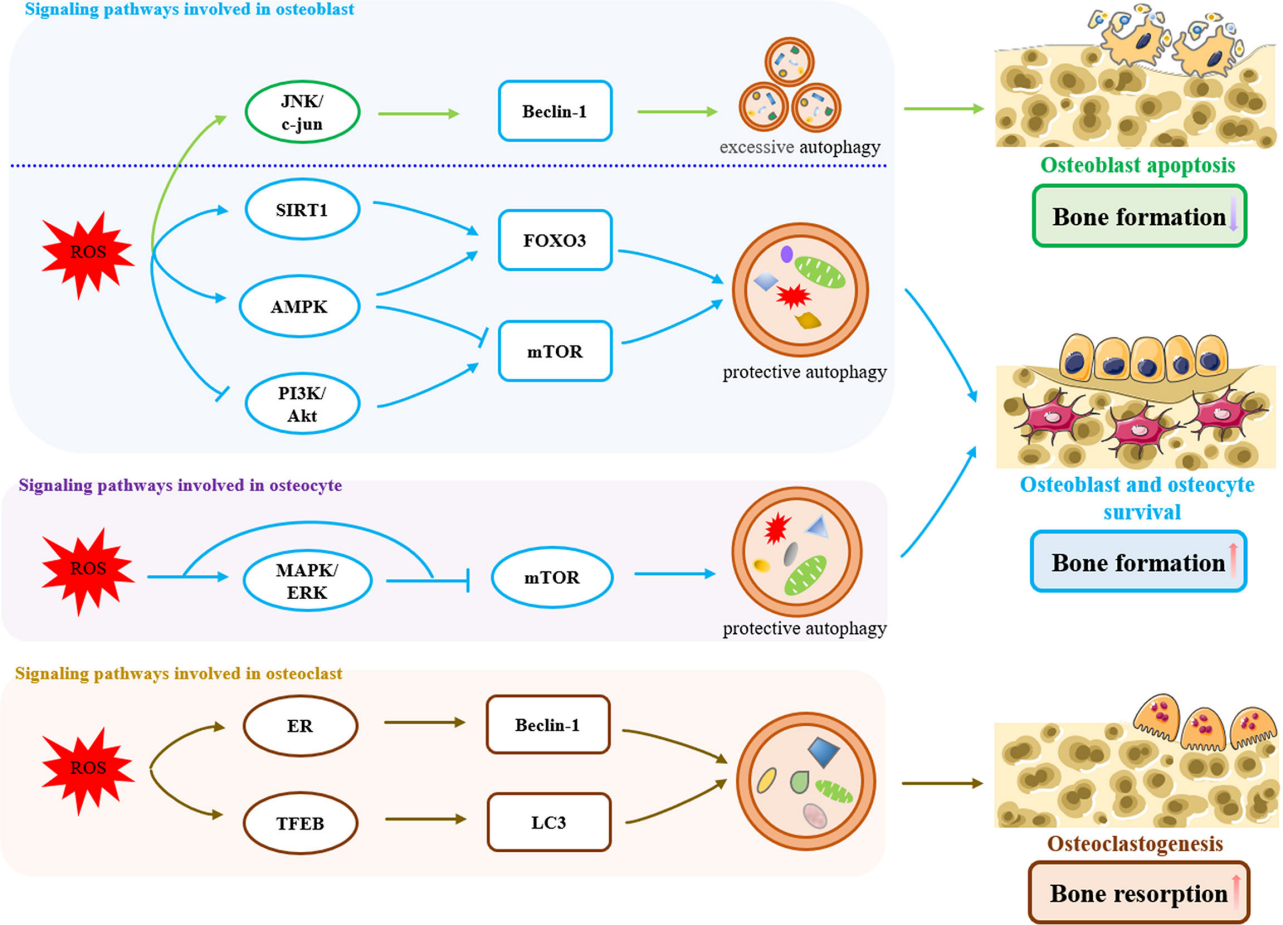
Signaling pathways involved in oxidative stress-induced autophagy in bone remodeling. In osteoblasts, ROS induced an excessive increase in Beclin-1 levels by activation of the JNK/c-jun pathway, which triggered excessive autophagy, exacerbated osteoblasts apoptosis, and reduced bone formation. On the other hand, oxidative stress activates protective autophagy through ROS/SIRT1/FOXO3, ROS/AMPK/FOXO3, ROS/AMPK/mTOR and ROS/PI3K/Akt/mTOR pathways to remove excessive ROS within a certain range, promoting the survival of osteoblasts and increasing bone formation. Likewise in osteoblasts, oxidative stress-induced protective autophagy is also present in osteocytes, which is achieved by ROS/MAPK/ERK/mTOR pathway. In osteoclasts, Oxidative stress-induced autophagy promotes osteoclastogenesis and bone resorption through the ROS/ER and ROS/TFEB pathways.

Oxidative stress is involved in the development of osteoporosis and aging, as evidenced by both ovariectomy and age-increased oxidative stress and reduction of the antioxidant system in rat femurs, which promotes the development of osteoporosis (65). As mentioned above, oxidative stress disrupts the balance of bone formation and resorption by inhibiting osteoblast function and promoting osteoclast activity. In response to oxidative stress-induced autophagy, ROS may act as an antioxidant during dysregulation of bone remodeling to protect from bone loss and osteoporosis. In the following, we describe the regulation of autophagy in response to oxidative stress in osteoblasts, osteocytes, and osteoclasts (**Figure1**).

### 4.1 Regulation of Autophagy in the Response to Oxidative Stress in Osteoblasts

A high glucose (HG) environment, glucocorticoids or estrogen deficiency cause a pathological increase in ROS levels, thereby impairing the osteoblast function (66–68). In response to ROS, autophagy is activated and promotes osteoblast function as a negative feedback loop. Alberto et al. found that HG increased protein oxidation and the ROS levels, thereby activating autophagy in MC3T3-E1 cells, which reduced damage from HG and protected the cells, whereas inhibition of autophagy increased cell apoptosis (66). In addition, increased ROS levels caused the LC3II/LC3I ratios to increase and p62/SQSTMI to decrease, as observed in advanced glycation end products (AGE)-treated osteoblasts. Furthermore, the autophagy agonist rapamycin (RA) attenuated AGE-induced apoptosis, while the autophagy inhibitor 3-methyladenine (3-MA) increased AGE-induced apoptosis, indicating that autophagy plays a critical role in protecting osteoblasts from AGE-induced apoptosis (69). Likewise, other studies have also demonstrated that osteoblast activity is regulated by glucocorticoids in a dose-dependent manner. Low doses of dexamethasone promoted osteoblast autophagy, protected from damage by ROS, and attenuated apoptosis in osteoblasts. However, as the dose and the duration of the dexamethasone treatment increased, the antioxidant effects of autophagy were overwhelmed, which then lead to apoptosis (67). These results reveal that the protective effect of ROS-induced autophagy is limited and dependent on the dose of ROS level and the duration of stimulation.

Osteoblasts experiencing stress from aging or ovariectomy in mice have been reported to have increased levels of ROS and swollen mitochondria, followed by a 95% decrease in LC3-II levels. Further research has indicated that osteoblast conditional autophagy deficiency in mice results in enhanced aging and estrogen deficiency-related bone loss (68). Conversely, estradiol administration has been shown to increase ULK1, Beclin1, and LC3II protein levels in osteoblasts, decrease oxidative stress levels, and significantly reduced the expression of apoptotic biomarkers through the ER-ERK-mTOR pathway (70). Thus, autophagy can be an important potential target for protection against damage from oxidative stress or ROS, but how autophagy responds to ROS signaling needs to be further explored. Following is a review of ROS/FOXO3, ROS/AMPK, ROS/Akt/mTOR, and ROS/JNK/c-Jun pathways that are involved in the autophagic response to oxidative stress in osteoblasts.

#### 4.1.1 ROS/FOXO3

Forkhead box O3(FOXO3) protein is a member of the FOXO family, which can be activated by catalase, SOD2, and glutathione peroxide in antioxidant reactions (71). In response to oxidative stress, MAPK8, MAPK14/p38α, and serine/threonine-protein kinase 4 (STK4)/MST1 phosphorylate FOXOs, causing their nuclear translocation as well as transcriptional activation of target genes such as manganese superoxide dismutase (MnSOD) and catalase (72, 73). During BMSC differentiation into osteoblasts, and then osteoblasts differentiation into osteocytes, the increasing level of ROS activates FOXO3 serine 294 phosphorylation, and FOXO3-induced autophagy then downregulates the increased ROS levels as a negative feedback loop to ensure proper differentiation (74). In addition, inhibition of MAPK11/12/14 kinase can reduce the nuclear translocation of FOXO3 by MSC exposure to oxidative stress, while LC3B and GABARAPL1 are significantly upregulated upon FOXO activation, suggesting that MAPK11/12/14 participate in the activation of FOXO3 by ROS and then activate autophagy. PARK2, a ubiquitin ligase that is indispensable for inducing mitochondrial autophagy, was also significantly increased when FOXO3 was induced by ROS, which is an important process for the clearance of ROS, while the process was impaired when FOXO3 was knocked down (74).

SIRT1 is another key factor involved in ROS-mediated FOXO3 activation. Gu et al. found that ROS/SIRT1/FOXO3 may be involved in the survival of the damage from fluoride in MC3T3-E1 osteoblasts. ROS-mediated activation of SIRT1 has been shown to increase the level of FOXO3 deacetylation and to promote the expression of its substrate Bnip3, which promotes the upregulation of autophagy levels and reduces fluoride-induced osteoblast apoptosis. Conversely, inhibition of SIRT1 expression has been shown to impair FOXO3-induced autophagy (75).

#### 4.1.2 ROS/AMPK

AMPK is a heterotrimeric complex comprising a catalytic subunit (α-subunit) and two regulatory subunits (β- and γ-subunits) (76). In addition to its role in energy metabolism, AMPK also acts as an oxidative stress sensor to regulate cell survival under stressful conditions (77). ROS activates AMPK by phosphorylating the AMPK alpha1 threonine 172 (78), and activated AMPK directly phosphorylates the mTORC1 subunit Raptor, which can then suppress the inhibitory effect of mTORC1 on ULK1 to promote autophagy. Moreover, AMPK also directly phosphorylates Ser 317 and Ser 777 of the UKL1 complex to activate autophagy (79). However, inhibition of autophagy enhances ROS-induced cell apoptosis. H_2_O_2_ can induce phosphorylation of ULK1 and upregulation of LC3B-II *via* activation of AMPK, while treatment with the autophagy inhibitors 3-MA and bafilomycin A1 increases H_2_O_2_-induced cell death. Furthermore, AMPKα knockdown has been reported to further inhibit ULK1 phosphorylation and LC3B-II upregulation, indicating that ROS/autophagy activation in osteoblasts requires AMPK, which can act as a negative feedback loop in the regulation of ROS levels when exposed to oxidative stress (80). Consistent with these results, the AMPK activators GSK621 or A-769662 enhance the protective autophagic response as evidenced by phosphorylation of ULK1 on Ser-317, upregulation of ATG5 and Beclin-1, and downregulation of p62 (81, 82) in case of H_2_O_2_-induced oxidative stress in osteoblasts.

#### 4.1.3 ROS/Akt/mTOR

The PI3K/Akt/mTOR pathway plays an essential role in stress responses, autophagy, cell survival, and apoptosis (83). The PI3K/Akt signaling axis activates mTOR by phosphorylation of p70S6K and 4EBP1, thereby inhibiting autophagy (84, 85). ROS initially regulate PI3K/Akt, and the PI3K/Akt pathway in turn regulates ROS homeostasis to promote cell survival (86). It has been reported that ROS levels are significantly elevated under high glucose conditions, and p-Akt and p-mTOR protein expression was significantly downregulated in MC3T3-E1 cells, while the antioxidant NAC reversed their expression and reduced osteoblasts apoptosis, suggesting that high levels of ROS promoted the protective autophagy by inhibition of the Akt/mTOR axis (87). Further study has revealed that the inactivation of phosphatase and tensin homologs (PTEN) when ROS activates PI3K may be the main reason for ROS inhibition of the Akt/mTOR signaling pathway, as PTEN inhibits the synthesis of PIP3 and thus activation of Akt signaling (88). The Chinese traditional medicine monotropein has been reported to protect against the damage from H_2_O_2_-induced oxidative stress in osteoblasts. Monotropein was found to decrease phosphorylation of Akt, mTOR, p70S6K, and 4EBP1, as well as upregulate Beclin-1 expression and LC3-II/LC3-I ratios, which then activated autophagy to increase osteoblastic bone formation (89). Monotropein, hence, appears to have potential for treatment or prevention of aging or estrogen-deficiency osteoporosis.

#### 4.1.4 ROS/JNK/c-Jun

Fluoride-mediated ROS triggers oxidative cell damage and apoptosis through N-terminal kinase (JNK)/c-Jun signaling. In contrast, the ROS-induced JNK/c-Jun pathway activates SIRT1 and triggers autophagy as an adaptive reaction to protect cells from fluoride damage (90). However, it has also been shown that the ROS-autophagy process mediated by the JNK pathway enhanced osteoblast apoptosis. Glucocorticoids upregulated JNK and c-Jun phosphorylation in osteoblasts, thereby activating JNK/c-Jun signaling pathway-induced autophagy, which then leads to increased apoptosis (91). ROS inhibitors have been reported to downregulate the JNK/c-Jun signaling pathway, but JNK inhibitors did not reduce ROS, indicating that ROS is an upstream signal for JNK, while autophagy and apoptosis occur in response to ROS/JNK/c-Jun signaling (91). Further studies have shown that JNK causes the degradation of the Beclin-1/Bcl-2 complex by phosphorylating Bcl2, and Beclin-1 excessively stimulates the onset of autophagy (92, 93), and a low level of Beclin-1 promotes autophagy for cell survival, while a high level of Beclin-1 induces autophagic cell death (94, 95). These findings indicated that JNK may be a potential target involved in the balance between oxidative stress-induced autophagy and apoptosis.

In the above studies, autophagy induced by oxidative stress may be a double-edged sword for osteoblasts. On the one hand, in response to aberrant ROS signaling, the MAPK/FOXO3, SIRT1/FOXO3, and AMPK pathways are activated, and the Akt/mTOR pathway is inhibited, leading to activation of autophagy and the scavenging of excessive ROS within a certain range, thereby promoting osteoblast survival and increasing bone formation. On the other hand, when ROS levels are so high as to exceed the clearance effect of protective autophagy, they can activate the JNK pathway and subsequently induce excessive autophagy, thereby enhancing apoptosis of osteoblasts and thus reducing bone formation. A large cascade of interdependent responses between autophagy and JNK-mediated apoptosis has been documented, but how the JNK pathway regulates the balance of autophagy and apoptosis in osteoblasts in response to ROS signaling remains to be fully elucidated.

### 4.2 Regulation of Autophagy in the Response to Oxidative Stress in Osteocytes

As in osteoblasts, oxidative stress-induced autophagy in osteocytes is also a protective response. Decreased estrogen levels are a prominent cause of postmenopausal osteoporosis. Yang et al. established an ovariectomized rat model that mimics the decrease in estrogen levels *in vivo*. They found a significant decrease in bone mineral density and bone mass in ovariectomized rats, accompanied by a decrease in antioxidant parameters such as the total antioxidant capacity, superoxide dismutase activity, catalase activity, and an increase in the expression level of osteocyte autophagy-related factors such as ATG5, LC3, and Beclin-1. In contrast, estrogen treatment prevented the decrease in bone mass and the abnormal increase in oxidative stress levels, and it restored autophagy to normal levels (96). These data suggest that estrogen deficiency can lead to an increase in oxidative stress levels *in vivo*, which in turn triggers its downstream protective autophagic response, but ultimately leads to the development of osteoporosis due to its limited protective effect. Further exploration of the negative feedback protection mechanism of autophagy in osteocytes has revealed that ROS/MAPK/ERK and ROS/mTOR/ULK1 signaling axes appear to play important roles (97, 98).

#### 4.2.1 ROS/MAPK/ERK

ERK is one of the classical signal transduction components of the MAPK family, and it can directly induce autophagy by upregulation of the expression of autophagy-related proteins such as LC3 and p62 (99). Rekha et al. found that treatment with low doses of glucocorticoids increased oxidative stress levels and basal autophagy levels in osteocytes without increasing osteocyte apoptosis, whereas high doses of glucocorticoids enhanced osteocyte apoptosis. Further studies have revealed that glucocorticoid treatment significantly increases MAPK and ERK phosphorylation in osteocytes, while the ERK-specific inhibitor U0126 completely abolished glucocorticoid-induced elevated LC3 expression. These data suggest that low-dose glucocorticoid-induced oxidative stress activates the MAPK/ERK signaling pathway, which in turn enhances autophagy levels and protects osteocytes from oxidative stress damage, whereas the protective effect of autophagy induced by high levels of glucocorticoids has a range and does not respond to abnormally elevated ROS levels, thus manifesting as excessive apoptosis of osteocytes (97).

#### 4.2.2 ROS/mTOR/ULK1

ULK1 is a key initiator protein in the induction of autophagy, and inhibition of mTOR activity can enhance autophagy levels by binding to and phosphorylating the serine site of ULK1 (100). Bisphenol A (BPA) is an environmental endocrine disruptor that can perturb bone metabolism and bone homeostasis (101). BPA has been reported to increase malondialdehyde and ROS levels in osteocytes and decrease the expression of the antioxidant enzymes nuclear factor E2-related factor 2 (Nrf2) and heme oxygenase-1 (HO-1), leading to oxidative stress. BPA has also been shown to significantly inhibited mTOR phosphorylation and promoted ULK1 phosphorylation, there inducing activation of autophagy. In contrast, treatment with the mTOR activator MHY1485 (MHY) or the ULK1 inhibitor SBI-0206965 (SBI) inhibited BPA-induced autophagy and enhanced apoptosis in osteocytes, but did not reduce ROS levels. Furthermore, NAC treatment attenuated the level of ROS-mediated autophagy. This suggests that the high level of ROS caused by BPA acts upstream of the mTOR/ULK1 signaling axis and that the autophagic response that it triggers is protective against the cytotoxic effects of BPA (98).

### 4.3 Regulation of Autophagy in the Response to Oxidative Stress in Osteoclasts

ROS acts as intracellular signaling mediators in osteoclast differentiation. RANKL stimulation of osteoclast precursor cells increases intracellular ROS production, and reduction of RANKL-induced ROS by NAC treatment down-regulates of MAPK, ERK, and other signaling pathways, thereby leading to attenuated osteoclast precursor differentiation (102, 103). Unlike autophagy acting as the cytoprotective role in osteoblasts, ROS-induced autophagy even promotes osteoclast differentiation and formation. High levels of ROS induced by glucocorticoids or inflammatory conditions act as a catalyst for osteoclastogenesis. Sul et al. found that lipopolysaccharide promoted autophagy and led to osteoclastogenesis by stimulating ROS production, while reduction of ROS by siNOX1 and siNOX2 dramatically diminished LC3II levels accumulation as well as the expression of osteoclast-specific genes expression (104). Interestingly, osteoclastogenesis was upregulated by glucocorticoids at high doses, but low doses had no effect (105). The accumulation of intracellular ROS in the presence of high glucocorticoid levels was synchronized with the upregulation of autophagic activity, which was prevented by the ROS scavenger NAC. While 3-MA administration blocked the promotion of osteoclast formation by glucocorticoids, it failed to reduce intracellular ROS accumulation. We further explored how ROS mediates autophagy to enhance osteoclastogenesis and we found that the ROS/ER and ROS/TFEB pathways may be involved in this process.

#### 4.3.1 ROS/ER

Endoplasmic reticulum stress (ER) is induced by the accumulation of misfolded proteins leading to an unfolded protein response. ROS can cause aggregation and misfolding of proteins (106). Activation of ER regulates autophagy, which in turn regulates cell survival and death (107). MCP-1 is an important protein in the differentiation of monocytes into osteoclast precursors, and p47PHOX expression and its membrane translocation expressions induced by MCP-1 have been reported to promote ROS production, which induced ER and subsequently promoted upregulation of the autophagy markers Beclin-1 and LC3II as well as expression of osteoclast-associated markers such as TRAP and Ctsk. 3-MA treatment or knockdown of Beclin-1 significantly suppressed TRAP and Ctsk expression without affecting ER or its upstream ROS levels (108). These results indicate that osteoclast precursor cell differentiation is mediated by ROS production, which leads to ER stress, thereby inducing autophagy and ultimately promoting osteoclastogenesis.

#### 4.3.2 ROS/TFEB

TFEB is a key transcription factor that controls the autophagy-lysosome system. Stress conditions such as lysosomal dysfunction or starvation cause nuclear translocation of TFEB and promote transcription of its target genes (109). ROS can directly oxidize TFEB, reduce its association with RRAG GTPase on lysosomes, and rapidly induce nuclear localization (110). Sul et al. found that high levels of ROS induced by 7-ketocholesterol (7-KC) significantly increased the nuclear translocation of TFEB and upregulated the lipidated form of LC3II in osteoclasts as well as the number and the activity of osteoclasts. In contrast, TFEB knockdown significantly downregulated autophagy levels and osteoclastogenesis. This suggests that 7-KC-mediated ROS induced oxidation of TFEB and promoted its nuclear translocation to enhance autophagy, leading to increased osteoclast numbers and activity (111).

## 5 Conclusion

We have provided an overview of the function of oxidative stress-mediated autophagy in bone remodeling. Oxidative stress-induced ROS impair bone formation by osteoblasts and osteocytes and promote bone resorption by osteoclasts, thereby disrupting the homeostasis of bone and enhancing the progression of osteoporosis. In addition, ROS also activates autophagy and then regulates osteoblasts and osteocytes in a negative feedback loop. However, ROS-mediated autophagy enhances osteoclast differentiation, which can overwhelm the protective effect in osteoblasts and osteocytes, as bone tissue exposed to oxidative stress leads to the development of osteoporosis. Therefore, further studies of the regulatory mechanisms of autophagy in redox signaling during pathological bone remodeling are needed. Furthermore, it may be possible to exploit the potential targets of autophagy for protective or therapeutic strategies against osteoporosis.

## Author Contributions

XC and LZ designed this review and supervised the whole program; CZ, SS and SZ searched the articles and offered advice; MH prepared the figure; CZ, SS and XC wrote the paper. All the authors reviewed and approved the manuscript.

## Funding

The work is supported by funding from Wenzhou basic scientific research project (Grant No. 2019Y0848) and the national undergraduate innovation and entrepreneurship training program (Grant No.202110343040).

## Conflict of Interest

The authors declare that the research was conducted in the absence of any commercial or financial relationships that could be construed as a potential conflict of interest.

## Publisher’s Note

All claims expressed in this article are solely those of the authors and do not necessarily represent those of their affiliated organizations, or those of the publisher, the editors and the reviewers. Any product that may be evaluated in this article, or claim that may be made by its manufacturer, is not guaranteed or endorsed by the publisher.
